# Targeting XPO1 enhances innate immune response and inhibits KSHV lytic replication during primary infection by nuclear stabilization of the p62 autophagy adaptor protein

**DOI:** 10.1038/s41419-020-03303-1

**Published:** 2021-01-04

**Authors:** Wen Meng, Shou-Jiang Gao

**Affiliations:** UPMC Hillman Cancer Center, Department of Microbiology and Molecular Genetics, University of Pittsburgh, Pittsburgh, PA USA

**Keywords:** Mechanisms of disease, Infection

## Abstract

Nucleocytoplasmic transport of signaling modulators is essential for regulating cellular responses to extracellular stimulation and stress, as well as pathogen infection. Exportin 1 (XPO1), also known as chromosomal maintenance 1 (CRM1), mediates nuclear export of proteins, rRNAs, snRNAs, and some mRNAs. In this study, we have identified an essential role of XPO1 in regulating Kaposi’s sarcoma-associated herpesvirus (KSHV) lytic replication during primary infection of primary human umbilical vein endothelial cells. Treatment with an XPO1 inhibitor KPT-8602 and short hairpin RNA (shRNA)-mediated knockdown of XPO1 reduced KSHV lytic replication but had no effect on KSHV entry and trafficking. XPO1 inhibition induced retention of autophagy adaptor protein p62 (SQSTM1) in the nucleus, which enhanced activation of TBK1 and IRF3. As a result, nuclear accumulation of p62 increased expression of innate immune-related genes including IRF7, ISG15, IFIT1, IFIT2, and IFIT3, leading to a reduction of KSHV lytic replication. These results illustrate a novel mechanism by which XPO1 mediates innate immune response and KSHV replication, and identify XPO1 as a potential therapeutic target and KPT-8602 as a promising therapeutic agent for KSHV infection.

## Introduction

Kaposi’s sarcoma-associated herpesvirus (KSHV) is a gammaherpesvirus etiologically associated with Kaposi’s sarcoma and several other lymphoproliferative diseases^1–3^. KSHV has both latent and lytic replication cycles regulated by intracellular and extracellular signals^4^. In lytic replication-permissive cells such as primary human umbilical vein endothelial (HUVEC), activation of ERK, p38, and JNK pathways promotes expression of viral lytic genes, and KSHV undergoes robust lytic replication before entering into latency^5–8^. However, activation of NF-κB pathway promotes the expression of latent genes and KSHV enters into latency in nonpermissive cells such as primary human dermal microvascular endothelial cells and human foreskin fibroblasts^9–12^.

The exchange of signaling molecules between nuclear and cytoplasmic compartments is essential for cellular functions. This process is mediated by a nuclear pore complex (NPC) inserted across the nuclear envelope (NE). Unlike smaller molecules, which diffuse through NPCs, the shuttling of proteins larger than 40 kDa is regulated by karyopherins (Kaps), which interact with NPC components nucleoporins (Nups) to cross NE^13^.

A Kap consists of a cargo-binding domain, an NPC-binding domain, and an N-terminal Ras-related nuclear protein (Ran) binding domain^14^. Ran is a small GTPase, switching between a cytoplasmic RanGDP form and a nuclear RanGTP form by nucleotide exchange and GTP hydrolysis. Kaps include both importins and exportins. For import, after importins transport cargo molecules through NPCs into the nucleus, RanGTP stimulates dissociation of the importin-cargo complex to release cargos^15^. For export, exportin-cargo complex formation is induced by RanGTP. After transportation into the cytoplasm, hydrolysis of GTP to GDP results in a switch from RanGTP to RanGDP to release cargos^16^.

There are seven members of exportin, including XPO1 (CRM1), XPO2 (CSE1L), XPO3 (XPOt), XPO4, XPO5, XPO6, and XPO7^13^. XPO1, XPO2, XPO4, XPO6, and XPO7 primarily mediate the export of proteins, whereas XPO3 and XPO5 are involved in the export of tRNAs and precursor microRNAs, respectively. XPO1 is the major exportin that mediates protein nuclear export, which is important for the replication of several viruses including retroviruses, orthomyxoviruses, paramyxoviruses, flaviviruses, coronaviruses, rhabdoviruses, and herpesviruses^17,18^. Hence, XPO1 is a potential target for antiviral therapy.

XPO1 transports proteins containing leucine-rich nuclear export signals (NESs)^19,20^. A novel class of selective inhibitor of nuclear export (SINE) compounds that bind to XPO1 NES groove was identified^21–23^. Unlike leptomycin B (LMB), SINE compounds form a reversible covalent bond with XPO1 cysteine 528, making it less cytotoxic than LMB to cells. KPT-8602 (Eltanexor) is a second-generation SINE with reduced penetration across blood–brain barrier and an enhanced tolerability profile^24^.

Here, we have identified a critical role of XPO1 in KSHV lytic replication during primary infection. We show that XPO1 inhibition with KPT-8602 and by shRNA knockdown significantly reduces infectious virions by inhibiting viral gene expression. XPO1 inhibition induces p62 nuclear retention, which blocks KSHV lytic replication by activating TBK1 and IRF3 and enhancing the expression of innate immune-related genes. These results identify XPO1 as an essential protein for KSHV lytic replication by regulating p62 nuclear shuttling and controlling antiviral immune responses.

## Materials and methods

### Cells and drug

HUVEC (ATCC) was cultured in VascuLife VEGF complete medium (Lifeline). KSHV-infected iSLK (iSLK-BAC16) cells were from Dr. Jae Jung and cultured as previously described^25^. KPT-8602 (Selleck) was dissolved in dimethyl sulfoxide (DMSO).

### Virus preparation and titration

KSHV was prepared from iSLK-BAC16 cells as previously described^25^. KSHV in VascuLife VEGF complete medium without Heparin was titrated by infecting HUVEC as previously described^5,26^.

### Virus infection

HUVEC were infected with KSHV at a multiplicity of infection of two IUs per cell^5,26^. To determine KPT-8602’s effect, HUVEC were pretreated with KPT-8602 or vehicle control DMSO for 1 h. Cells were then infected with KSHV with KPT-8602 or vehicle. At 4 h post infection (hpi), the medium was replaced with fresh medium. Cells were fixed at 6 hpi for examining virus entry and trafficking, at 24 hpi for examining viral infectivity, and at 48 hpi for examining viral transcripts and proteins. To examine infectious virions, supernatants were collected at 96 hpi and titrated^5,26^.

### Immunofluorescence assay

Immunofluorescence assay was performed as previously described^5^ using antibodies to ORF65^5^, LANA (Abcam), p62 (Cell Signaling), phosphorylated IRF3 (pIRF3, Novus Biologicals), and pTBK1 (Cell Signaling). Signal was detected with an Alexa488-, Alexa555-, or Alexa647-conjugated secondary antibody (Life Technologies). Images were acquired using an Olympus FV2000 Confocal Microscope with CellSens Software. Magenta of Alexa647 was shown as green pseudocolor for better presentation.

### Western blotting

Western blotting was performed as previously described^27^ using primary antibodies against LANA (Abcam), K-bZIP (Santa Cruz), β-actin (Santa Cruz), XPO1 (Sigma), p62 (Cell Signaling), pTBK1 (Cell Signaling), TBK1 (Novus Biologicals), pIRF3 (Novus), IRF3 (Novus), STING (Novus), and LC3B (Sigma). ORF65 and RTA antibodies were previously described^5,28^.

### RNA extraction and RT-qPCR

Expression of viral genes was analyzed by reverse transcription-quantitative polymerase chain reaction (RT-qPCR) as previously described^8^. qPCR primers were listed in Supplementary Table 1. Three biological repeats were performed for each treatment.

### DNA extraction

DNA extraction was performed by using QIAamp DNA Mini Kit (Qiagen).

### shRNA knockdown

Lentiviral production and knockdown with shRNAs were performed as previously described^29^. Supernatants were collected 72 h after transfection. HUVEC were infected with lentiviruses by spinning infection at 500 × *g* in 10 µg/ml Polybrene (Sigma). The effect of shRNA knockdown was examined at 48 hpi.

### Statistical analysis

Results are expressed as means and standard deviations. Statistical analyses were performed using Prism 5.01 (GraphPad Software). Significance was determined by two-way ANOVA followed by Tukey’s multiple comparisons test.

### Ethics statement

All authors had access to all data and have reviewed and approved the final paper.

## Results

### XPO1 inhibitor KPT-8602 decreases KSHV titer

**Fig. 1:**
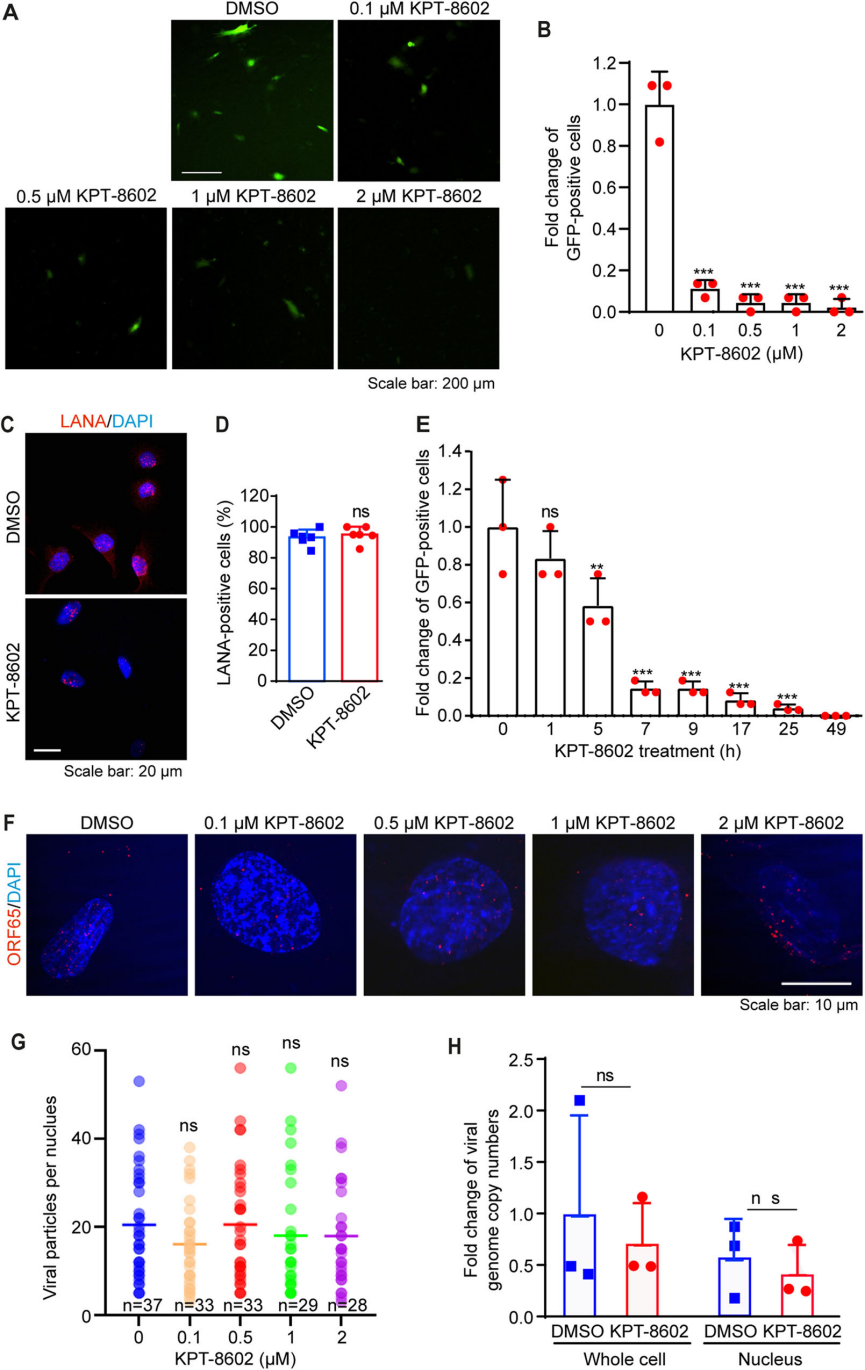
XPO1 inhibitor KPT-8602 decreases the production of KSHV infectious virions but has no effect on KSHV infectivity, and virus entry and trafficking during primary infection. **A**, **B** HUVEC were pretreated with the indicated doses of KPT-8602 for 1 h and then infected with KSHV in the presence of KPT-8602. At 4 hpi, cells were extensively washed and replaced with a new medium containing the indicated concentrations of KPT-8602. At 48 hpi, cells were extensively washed and replaced with a new medium without KPT-8602. Supernatants were collected at 96 hpi and used to titrate infectious virions by infecting fresh HUVEC. At 48 hpi, representative images were taken (**A**) to quantify the number of GFP-positive cells (**B**). **C** HUVEC pretreated with 0.5 µM KPT-8602 for 1 h were infected with KSHV in the presence of KPT-8602. At 48 hpi, cells were fixed and stained for LANA (red). The expression level and staining pattern of LANA are shown. Nuclei were stained with DAPI. Images were taken with confocal microscopy (magnification ×600). **D** Quantification of LANA-positive cells based on five images taken as described in (**C**). **E** HUVEC were pretreated with 0.5 µM KPT-8602 for 1 h and then infected with KSHV in the presence of KPT-8602 for the indicated times. Cells were extensively washed and replaced with a new medium without KPT-8602. At 50 hpi, cells were replaced with the new medium. Supernatants were collected at 96 hpi and used to titrate infectious virions by infecting fresh HUVEC. At 48 hpi, GFP-positive cells were quantified. **F** HUVEC pretreated with 0.5 µM KPT-8602 for 1 h were infected with KSHV in the presence of KPT-8602 for 6 h, fixed, and stained for nuclei with DAPI (blue) and KSHV particles with an antibody against ORF65 (red). Images were taken with a confocal microscopy (magnification ×1000). **G** The total number of KSHV particles successfully docked at the perinuclear region was determined and analyzed based on images taken as described in (**F**). **H** HUVEC pretreated with 0.5 µM KPT-8602 for 1 h were infected with KSHV in the presence of KPT-8602. Cells were harvested at 6 hpi. Cellular DNA and viral DNA were extracted from whole cells or the nuclear fraction for the detection of the viral genome by qPCR using LANA-specific primers. *, **, and *** indicate *P* values of <0.05, < 0.01, and <0.001, respectively; NS not significant.

We used KPT-8602 to investigate XPO1’s role in KSHV primary infection in HUVEC as these cells support productive infection^5^. HUVEC treated with KPT-8602 for 1 h were infected with KSHV. At 48 hpi, cells were replaced with fresh medium without KPT-8602. At 96 hpi, supernatants were collected and titrated. KPT-8602 reduced KSHV titer at a dose-dependent fashion (Fig. 1A, B). KPT-8602 reduced titers by 84% at 0.1 µM and by 98% at 2 µM. At 0.1 µM, KPT-8602 had <30% cytotoxic effect on cells before 48 h, and >40% of the cells remained alive at 72 and 96 h (Supplementary Fig. 1). At 2 µM, >45% of the cells remained viable at any points examined. Thus, KPT-8602’s inhibitory effect on the production of KSHV virions was unlikely due to its cell cytotoxicity.

### KPT-8602 inhibits KSHV lytic replication at post-entry stage(s)

To identify step(s) of KSHV infection regulated by XPO1, we examined whether XPO1 inhibition might affect infectivity. We used 0.5 µM KPT-8602 based on the cytotoxicity results (Supplementary Fig. 1). Over 70% of cells survived at 24 and 48 h, and >40% of cells survived at 72 and 96 h. HUVEC pretreated with DMSO or 0.5 µM KPT-8602 for 1 h were infected with KSHV. At 4 hpi, cells were replaced with fresh medium without the inhibitor. At 48 hpi, cells were stained for LANA protein. Detection of LANA protein indicated successful viral infection. KPT-8602 treatment did not affect the percentage of LANA-positive cells (Fig. 1C, D), LANA staining pattern and intensity, and the number of LANA punctate dots per cell (Fig. 1C). Hence, KPT-8602 neither affected KSHV infectivity nor LANA protein expression. To confirm that KPT-8602 was unlikely to affect KSHV entry and trafficking, we treated HUVEC with 0.5 µM KPT-8602 for 1 h and then infected them with KSHV. Cells were replaced with fresh medium without the inhibitor at the indicated time points. Supernatants were titrated for infectious virions at 96 hpi. KPT-8602 treatment for 1 h had no significant effect on KSHV titer (Fig. 1E). However, KPT-8602 treatment for >5 h significantly reduced KSHV titer in a time-dependent fashion (Fig. 1E). Particularly, KPT-8602 treatment for >7 h reduced KSHV titer by 84%. These results confirmed that KPT-8602 was unlikely to affect the early steps of KSHV infection.

We then investigated whether KPT-8602 affected the trafficking of KSHV particles to the nucleus. HUVEC pretreated with DMSO or KPT-8602 for 1 h were infected with KSHV for 6 h and stained for ORF65 protein to directly visualize viral capsids (Fig. 1F)^30^. KPT-8602 treatment did not affect the numbers of viral particles reaching the nucleus (Fig. 1F, G). We further examined KSHV genomes injected into the nucleus by qPCR. HUVEC pretreated with DMSO or 0.5 µM KPT-8602 for 1 h were infected with KSHV with KPT-8602. At 6 hpi, the nuclear and cytoplasmic separation was performed, and both cellular and viral DNA were extracted and quantified by qPCR. XPO1 inhibition did not affect the numbers of viral genomes in cells and in the nucleus (Fig. 1H). These results confirmed that KPT-8602 had no effect on virus entry and trafficking. Thus, KPT-8602 likely reduced KSHV virion production at a post entry stage.

### KPT-8602 inhibits expression of KSHV lytic genes

**Fig. 2:**
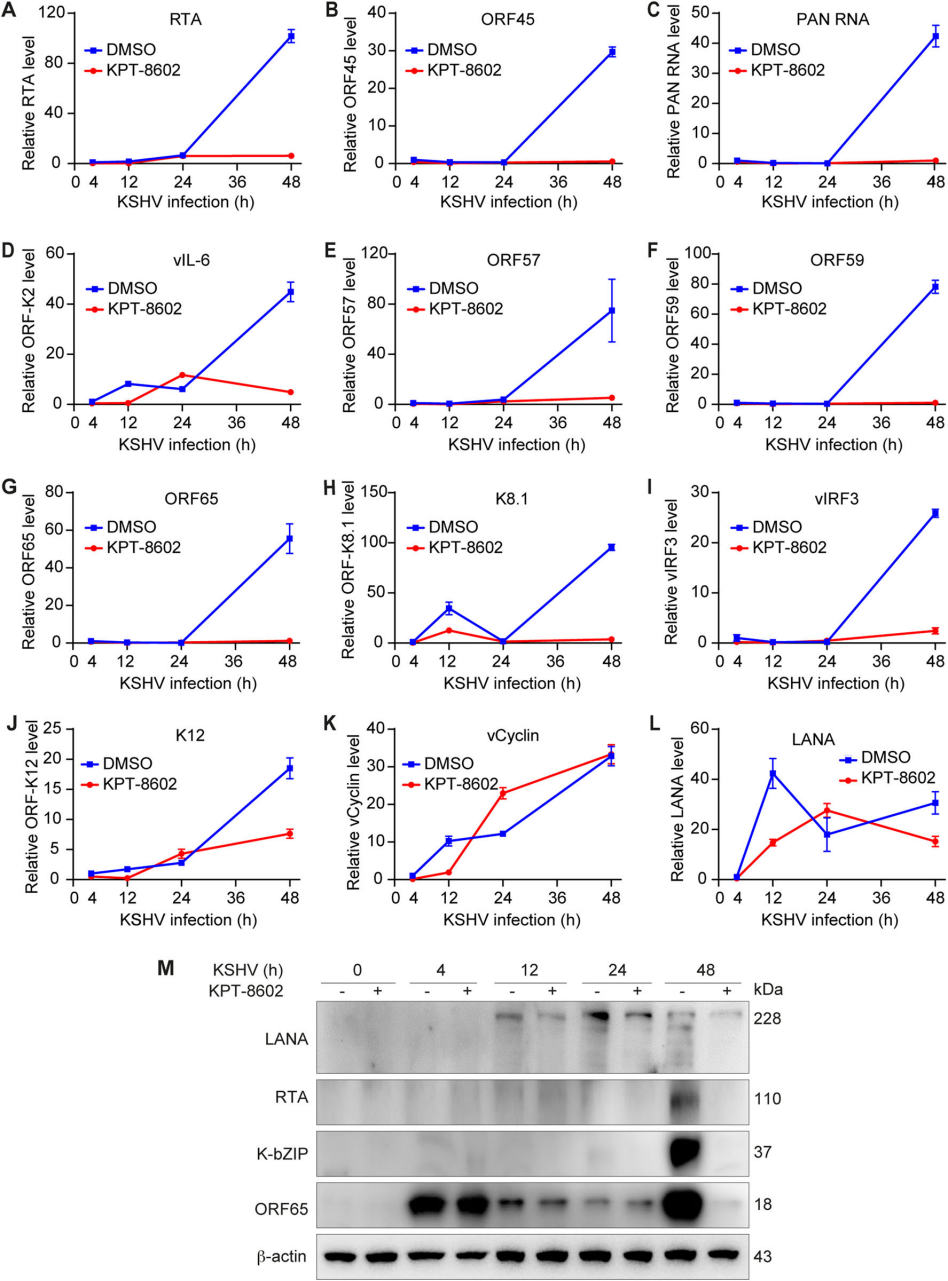
XPO1 inhibitor KPT-8602 inhibits the expression of KSHV genes except for LANA and vCyclin genes during primary infection. **A**–**L** HUVEC were pretreated with 0.5 µM KPT-8602 for 1 h and then infected with KSHV in the presence of KPT-8602 for 4, 12, 24, 36, and 48 h. Cells were collected for RNA extraction and examined for the expression of KSHV transcripts by RT-qPCR. **M** HUVEC were pretreated with 0.5 µM KPT-8602 for 1 h and then infected with KSHV in the presence of KPT-8602. The cell lysate was collected at 4, 12, 24, and 48 hpi and analyzed for the expression of viral proteins by Western blotting. β-actin served as the loading control.

KSHV lytic genes include immediate-early (IE), early (E), and late (L) genes, whereas latent genes mainly consist of LANA, vFLIP, vCyclin, ORF-K12, and a cluster of microRNAs^4^. During KSHV primary infection in HUVEC, latent transcripts expressed first, followed by lytic transcripts in the order of IE, E, and L transcripts, which peaked at around 54 hpi^8^. We monitored the kinetics of KSHV lytic genes by RT-qPCR. KPT-8602 significantly reduced mRNA levels of lytic genes including RTA (ORF50), ORF45, vIL-6 (ORF-K2), MTA (ORF57), ORF59, ORF65, and ORF-K8.1 (Fig. 2A–H). For KSHV latent genes, KPT-8602 treatment also inhibited the expression of vIRF3 and ORF-K12 (Fig. 2I, J) but had no significant effect on vCyclin and LANA (Fig. 2K, L). The Western-blotting analysis confirmed that KPT8602 treatment reduced the expression of lytic proteins RTA, K-bZIP (ORF-K8), and ORF65 but only had a marginal effect on latent protein LANA (Fig. 2M), which was consistent with LANA’s immunostaining results (Fig. 1C, D). These results demonstrated that XPO1 had an essential role in the expression of KSHV lytic genes.

### XPO1 knockdown inhibits KSHV lytic replication

**Fig. 3:**
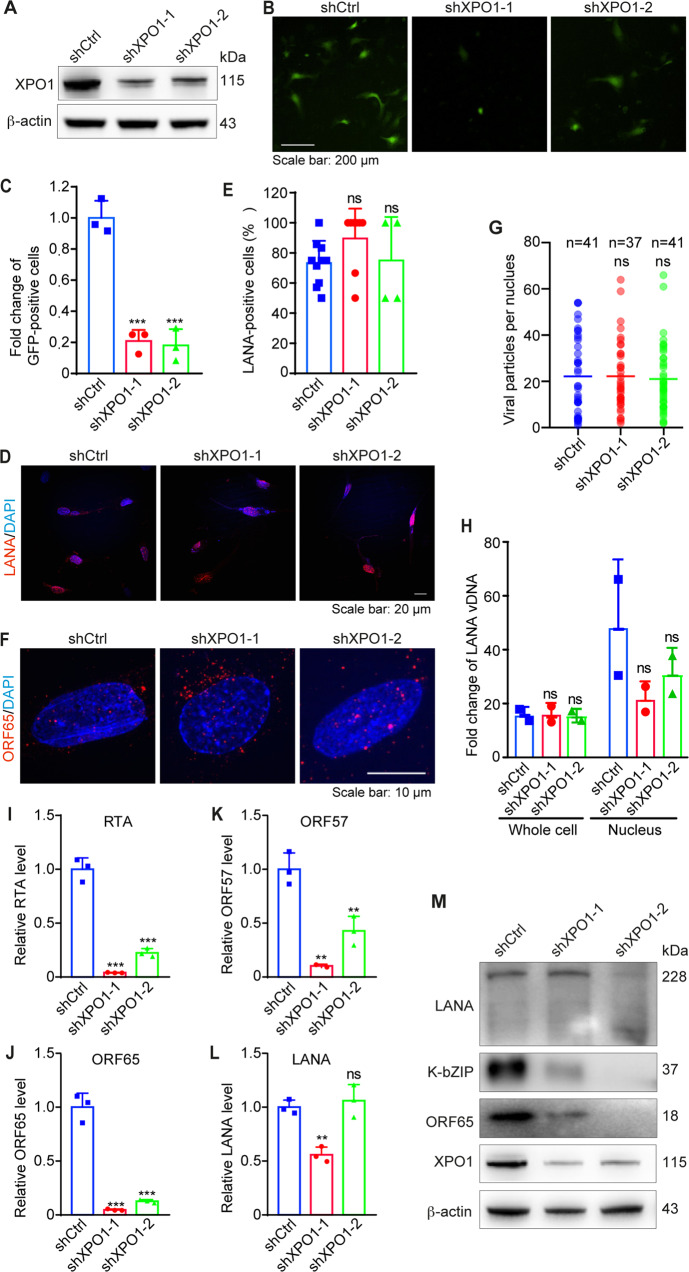
XPO1 knockdown blocks the KSHV lytic replication program but has no effect on KSHV infectivity, and virus entry and trafficking during primary infection. **A** HUVEC were infected with lentiviruses expressing shRNAs targeting XPO1 (shXPO1-1 and shXPO1-2) or control shRNA (shCtrl). Cells were selected with puromycin (1 µg/ml) for 48 h and analyzed by Western-blotting to monitor the knockdown efficiency. β-actin was used as the loading control. **B**, **C** HUVEC with XPO1 knockdown were infected with KSHV for 96 h. Supernatants were collected and used to titrate infectious virions by infecting fresh HUVEC. At 48 hpi, representative images were taken (**B**) and used for quantifying the numbers of GFP-positive cells (**C**). **D** HUVEC with XPO1 knockdown were infected with KSHV for 48 h. Cells were fixed and stained for LANA (red). The expression level and pattern of LANA are shown. Nuclei were stained with DAPI. Images were taken with a confocal microscopy (magnification ×600). **E** Quantification of LANA-positive cells based on images taken as described in (**D**). **F** HUVEC with knockdown of XPO1 were infected with KSHV for 6 h, fixed, and stained for nuclei with DAPI (blue) and KSHV particles with an antibody to ORF65 (red). Images were taken with a confocal microscopy (magnification ×1000). **G** The total number of KSHV particles successfully docked at the perinuclear region of each nucleus was quantified and analyzed based on images taken as described in (**F**). **H** HUVEC with XPO1 knockdown infected with KSHV for 6 h were collected and analyzed for viral genome copy numbers by qPCR using LANA primers. **I**–**L** HUVEC with XPO1 knockdown were infected with KSHV and then collected for RNA extraction at 48 hpi. The expression levels of KSHV transcripts were then examined by RT-qPCR. **M** HUVEC with XPO1 knockdown were infected with KSHV and collected at 48 hpi. The expression of viral proteins was then analyzed by Western blotting. β-actin was used as a loading control. *, **, and *** indicate *P* values of < 0.05, < 0.01, and < 0.001, respectively; NS not significant.

To confirm KPT-8602’s inhibitory effect on KSHV replication, we performed shRNA knockdown of XPO1. XPO1 protein was reduced by >80% in HUVEC transduced with lentiviruses expressing XPO1 shRNAs (Fig. 3A). XPO1 knockdown significantly reduced infectious virions by 80% compared to the scrambled control (Fig. 3B, C), confirming KPT-8602’s effect and XPO1’s critical role in KSHV replication.

To confirm the step of KSHV replication regulated by XPO1, we determined the effect of XPO1 knockdown on KSHV infectivity. HUVEC with XPO1 knockdown were infected with KSHV and stained for LANA protein at 48 hpi. XPO1 knockdown did not change the percentage of LANA-positive cells (Fig. 3D, E), and LANA staining pattern and intensity (Fig. 3D), indicating that XPO1 neither regulated KSHV infectivity nor LANA protein expression.

We further examined the effect of XPO1 knockdown on KSHV entry and trafficking. HUVEC with XPO1 knockdown were infected with KSHV for 6 h and stained for ORF65 protein (Fig. 3F). There was no difference in virus particles docked at the perinuclear region between cells transduced with XPO1 shRNAs or scrambled control (Fig. 3G). XPO1 knockdown also did not affect the internalization of the viral genome into cells and the nucleus (Fig. 3H). These results confirmed that XPO1 did not regulate virus entry and trafficking.

Finally, we investigated the effect of XPO1 knockdown on the expression of KSHV genes. HUVEC with XPO1 knockdown were infected with KSHV for 48 h. XPO1 knockdown significantly reduced mRNA levels of lytic genes RTA, ORF57, and ORF65 (Fig. 3I–K) but only had a minor effect on latent gene LANA (Fig. 3L). XPO1 knockdown also significantly decreased lytic proteins K-bZIP and ORF65 but not latent protein LANA (Fig. 3M). These results confirmed XPO1’s essential role in KSHV lytic replication during primary infection.

### KPT-8602 retains p62 in the nucleus and enhances IRF3 and TBK1 activation

We have previously shown that p62 protein is retained in the nucleus in AGS and HUH7 cells following KPT-8602 treatment^31^. KPT-8602 treatment upregulated p62 protein level and retained p62 in nucleus of uninfected HUVEC (Supplementary Fig. 2). KSHV infection only had a marginal effect on the p62 level.

**Fig. 4:**
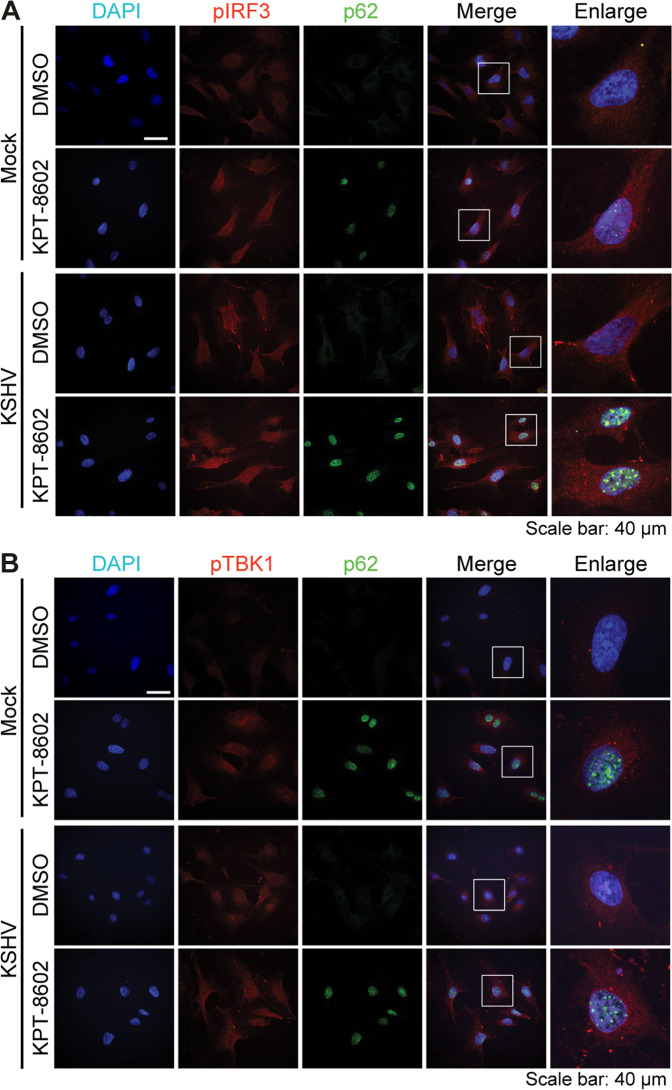
Treatment with XPO1 inhibitor KPT-8602 induces p62 nuclear retention and enhances IRF3 and TBK1 activation. **A**, **B** HUVEC were pretreated with KPT-8602 at 0.5 µM or vehicle control DMSO for 1 h and then infected with KSHV for 12 h in the presence of KPT-8602 or DMSO. Cells were fixed and co-stained for pIRF3 (**A**) or pTBK1 (**B**) together with p62. Nuclei were stained with DAPI. Images were taken with confocal microscopy (magnification ×600). Magenta p62 was pseudo-colored to green for better observation.

It was reported that cytosolic p62 could attenuate the cGAS-STING pathway and RIG-mediated type I interferon (IFN) signaling^32,33^. We investigated the activation of TBK-1 and IRF3 upon KPT-8602 treatment. HUVEC were pretreated with 0.5 µM of KPT-8602 or DMSO for 1 h, and then mock-infected or infected with KSHV. At 12 hpi, KSHV infection slightly activated both TBK-1 and IRF3 (Fig. 4A, B) as previously reported^34,35^. While infection by a DNA virus can activate an innate immune response, KSHV encodes numerous genes that antagonize this response^34,36–40^. KPT-8602 treatment alone was sufficient to increase levels of both pIRF3 and pTBK1 (Fig. 4A, B). Hence, KPT-8602-induced p62 nuclear accumulation and stabilization might lead to enhanced activation of TBK1 and IRF3.

### XPO1 knockdown upregulates p62 expression and enhances type I IFN signaling

**Fig. 5:**
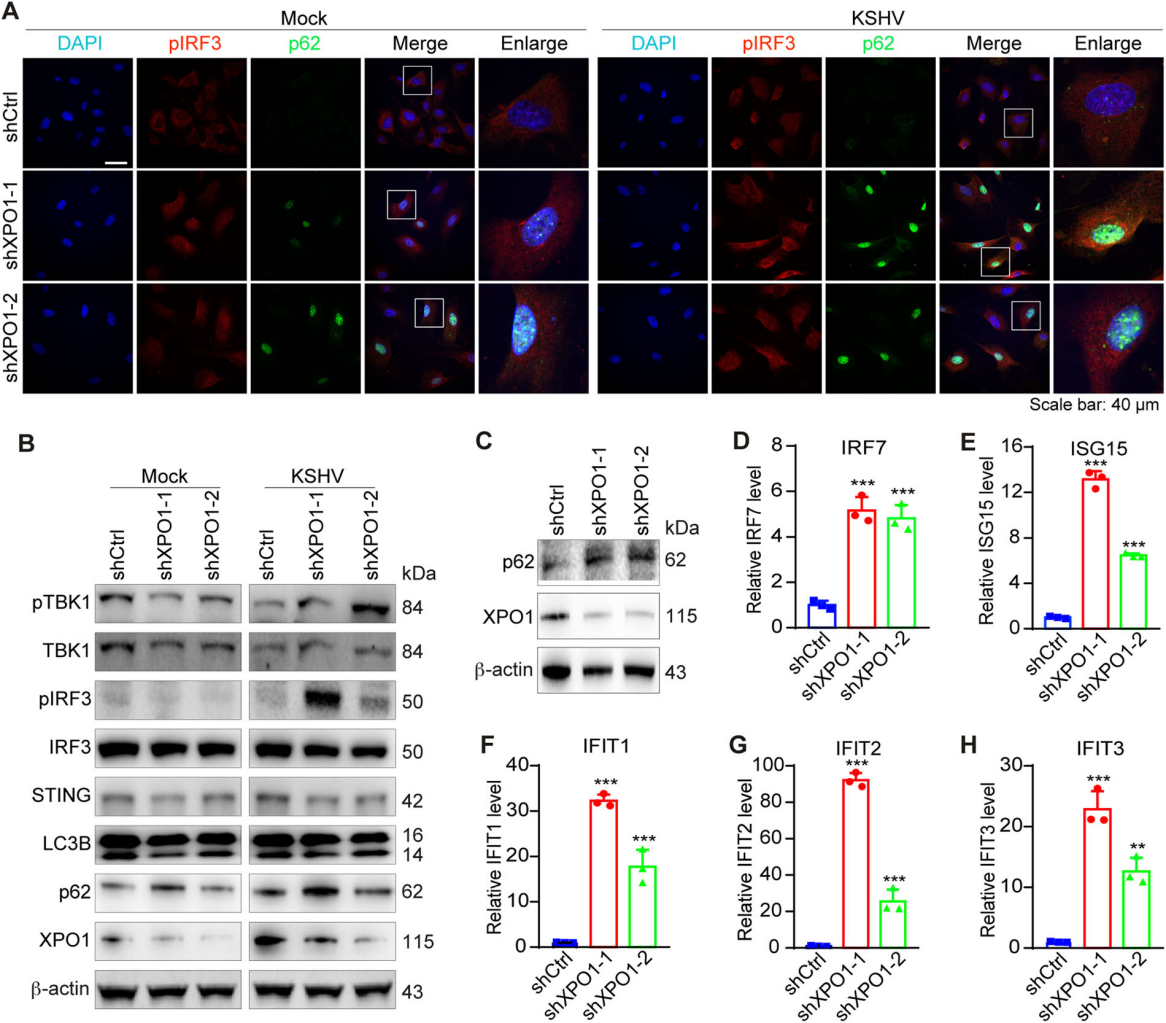
XPO1 knockdown induces p62 nuclear accumulation, enhances IRF3 and TBK1 activation, and upregulates the expression of immune-related genes. **A** HUVEC with XPO1 knockdown were infected with KSHV for 12 h, fixed and co-stained for pIRF3 and p62. Nuclei were stained with DAPI. Images were taken with a confocal microscopy (magnification ×600). Magenta p62 was presented as green pseudocolor for better observation. **B** HUVEC with XPO1 knockdown were infected with KSHV. Cell lysate was collected at 12 hpi and analyzed for levels of pTBK1, TBK1, pIRF3, IRF3, STING, LC3B, p62, and XPO1 by Western-blotting. β-actin was used as a loading control. **C** HUVEC with XPO1 knockdown were infected with KSHV for 48 h. Cell lysates were collected and analyzed for the expression of p62 by Western-blotting. β-actin was used as a loading control. **D**–**H** HUVEC with XPO1 knockdown were infected with KSHV for 48 h. Cells were collected for RNA extraction and examined for the expression of innate immune-related genes IRF7 (**D**), ISG15 (**E**), IFIT1 (**F**), IFIT2 (**G**), and IFIT3 (**H**) by RT-qPCR. *, **, and *** indicate *P* values of < 0.05, < 0.01, and < 0.001, respectively; NS not significant.

Next, we confirmed XPO1’s role in the innate immune response by performing XPO1 knockdown. Similar to KPT-8602 treatment, XPO1 knockdown resulted in p62 nuclear accumulation (Supplementary Fig. 3), increased pIRF3 level, and enhanced KSHV activation of pIRF3 and pTBK1 (Fig. 5A, B). However, XPO1 knockdown did not affect STING protein level. There was no obvious change of LC3B cleavage or p62 level following XPO1 knockdown (Fig. 5B), indicating that XPO1 knockdown did not affect autophagy flux and STING was unlikely degraded by p62-mediated autophagy. These results indicated that the enhanced TBK1 and IRF3 activation by XPO1 inhibition were unlikely due to p62’s cytosolic function, rather it might be the result of p62 nuclear accumulation.

To determine whether the increased TBK1 and IRF3 activation led to enhanced innate immune responses following XPO1 knockdown, we examined innate immune-related genes. IFIT1, IFIT2, and IFIT3, which play important roles in KSHV lytic replication^41^. Compared to control cells, XPO1 knockdown significantly increased mRNA levels of IRF7, ISG15, IFIT1, IFIT, and IFIT3 at 48 hpi in KSHV-infected HUVEC (Fig. 5C–H). These results indicated that XPO1 inhibition indeed enhanced innate immune responses.

### p62 knockdown restores KSHV lytic replication program but has no effect on virus entry, trafficking, and infectivity

**Fig. 6:**
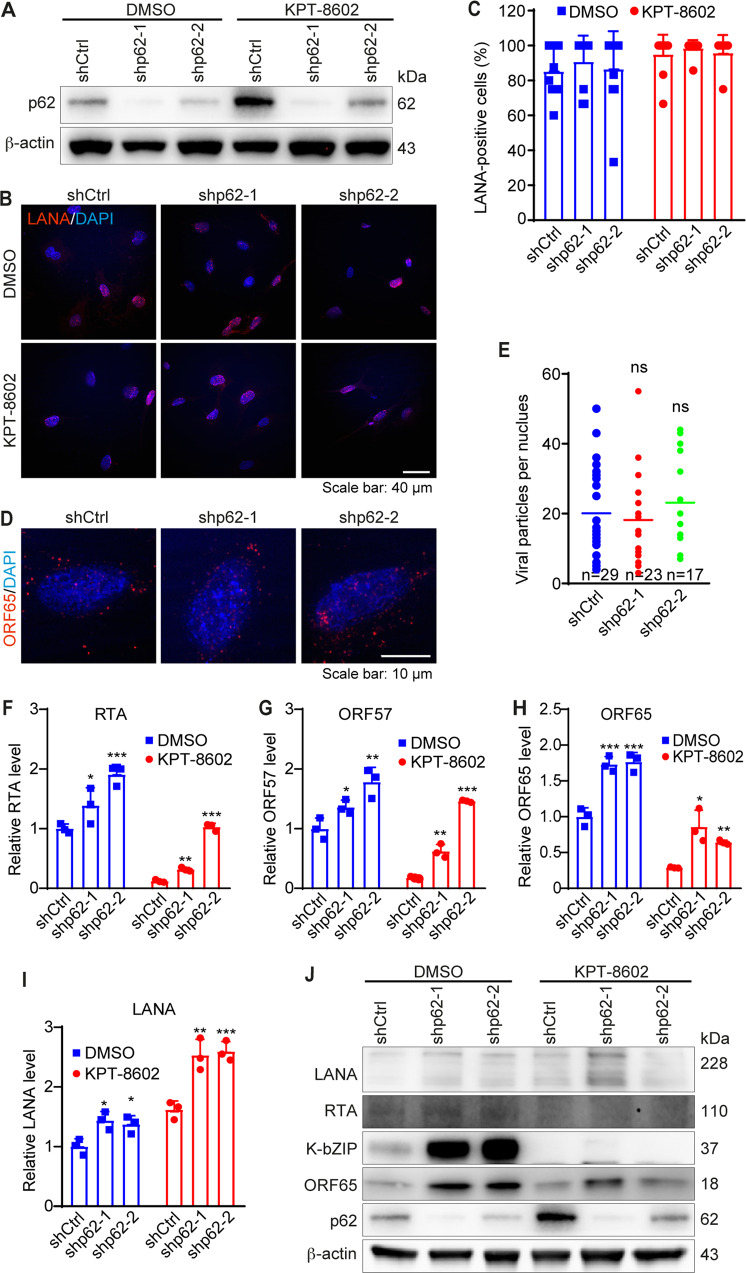
p62 knockdown reverses the inhibitory effect of XPO1 inhibitor KPT-8602 on KSHV lytic replication but has no effect on virus infectivity, and entry and trafficking during primary infection. **A** HUVEC were infected with lentiviruses expressing shRNAs targeting p62 (shp62-1 and shp62-2) or a control shRNA (shCtrl). Cells were selected with puromycin (1 µg/ml) for 48 h and analyzed by Western-blotting to monitor the knockdown efficiencies. β-actin served as the loading control. **B** HUVEC with p62 knockdown were pretreated with 0.5 µM KPT-8602. Cells infected with KSHV for 48 h in the presence of KPT-8602 (0.5 µM) were fixed and stained for LANA (red). The expression level and staining pattern of LANA protein are shown. Nuclei were stained with DAPI. Images were taken with a confocal microscopy (magnification ×600). **C** Quantification of LANA-positive cells using images taken as described in (**B**). **D** HUVEC with p62 knockdown were pretreated with 0.5 µM KPT-8602 for 1 h, and then infected with KSHV in the presence of KPT-8602 (0.5 µM) for 6 h. Cells were fixed and stained for nuclei with DAPI (blue) and KSHV particles with an antibody to ORF65 (red). Images were taken with a confocal microscopy (magnification ×1000). **E** The total number of KSHV particles successfully docked at the perinuclear region of each nucleus was quantified and analyzed based on images taken as described in (**D**). **F**–**I** HUVEC with p62 knockdown was pretreated with 0.5 µM KPT-8602 for 1 h and then infected with KSHV in the presence of KPT-8602 (0.5 µM). Cells were collected for RNA extraction at 48 hpi and examined for the expression of KSHV genes including RTA (**F**), ORF57 (**G**), ORF65 (**H**), and LANA (**I**) by RT-qPCR. **J** HUVEC with p62 knockdown were pretreated with 0.5 µM KPT-8602 for 1 h and then infected with KSHV for 48 h in the presence of KPT-8602 (0.5 µM). Cell lysate was collected and analyzed for the expression of p62 and KSHV proteins by Western-blotting. β-actin served as the loading control. *, **, and *** indicate *P* values of < 0.05, < 0.01, and < 0.001, respectively; NS not significant.

Our results so far showed that XPO1 inhibition reduced KSHV lytic replication by inhibiting the expression of viral lytic genes, which was correlated with p62 nuclear accumulation and enhanced innate immune responses. We further examined p62’s role in KSHV primary infection. p62 knockdown had no effect on KSHV infectivity as there was no obvious change in LANA-positive cells with or without KPT-8602 treatment (Fig. 6A–C). p62 knockdown also had no effect on LANA staining pattern and intensity (Fig. 6B), and the number of KSHV particles docked at the perinuclear region (Fig. 6D, E). These results indicated that p62 had no effect on KSHV entry, trafficking, and infectivity. However, p62 knockdown significantly increased mRNA levels of lytic genes RTA, Kb-ZIP, and ORF65 in the presence of KPT-8602, and rescued the inhibitory effect of KPT-8602 on these viral genes (Fig. 6F, H), indicating that p62 regulated expression of KSHV lytic genes. Interestingly, latent gene LANA was also increased, which persisted in the presence of KPT-8602 despite it had no noticeable effect on LANA mRNA (Fig. 6I), suggesting possible different mechanisms regulating KSHV genes following p62 knockdown and KPT-8602 inhibition. p62 knockdown also significantly increased levels of KSHV RTA, Kb-ZIP, and ORF65 proteins (Fig. 6J). Because of KPT-8602’s strong inhibitory effect on lytic proteins, the enhanced effect of p62 knockdown was not obvious for RTA and Kb-ZIP but was still visible for ORF65 protein (Fig. 6J). Consistent with the mRNA results, p62 knockdown also slightly increased LANA protein level without KPT-8602 treatment albeit it was less consistent with KPT-8602 treatment (Fig. 6J). These results indicated that increased p62 nuclear accumulation might at least partially mediate the inhibitory effect of XPO1 inhibition on KSHV lytic genes.

### p62 nuclear accumulation induces an innate immune response to block KSHV lytic replication

**Fig. 7:**
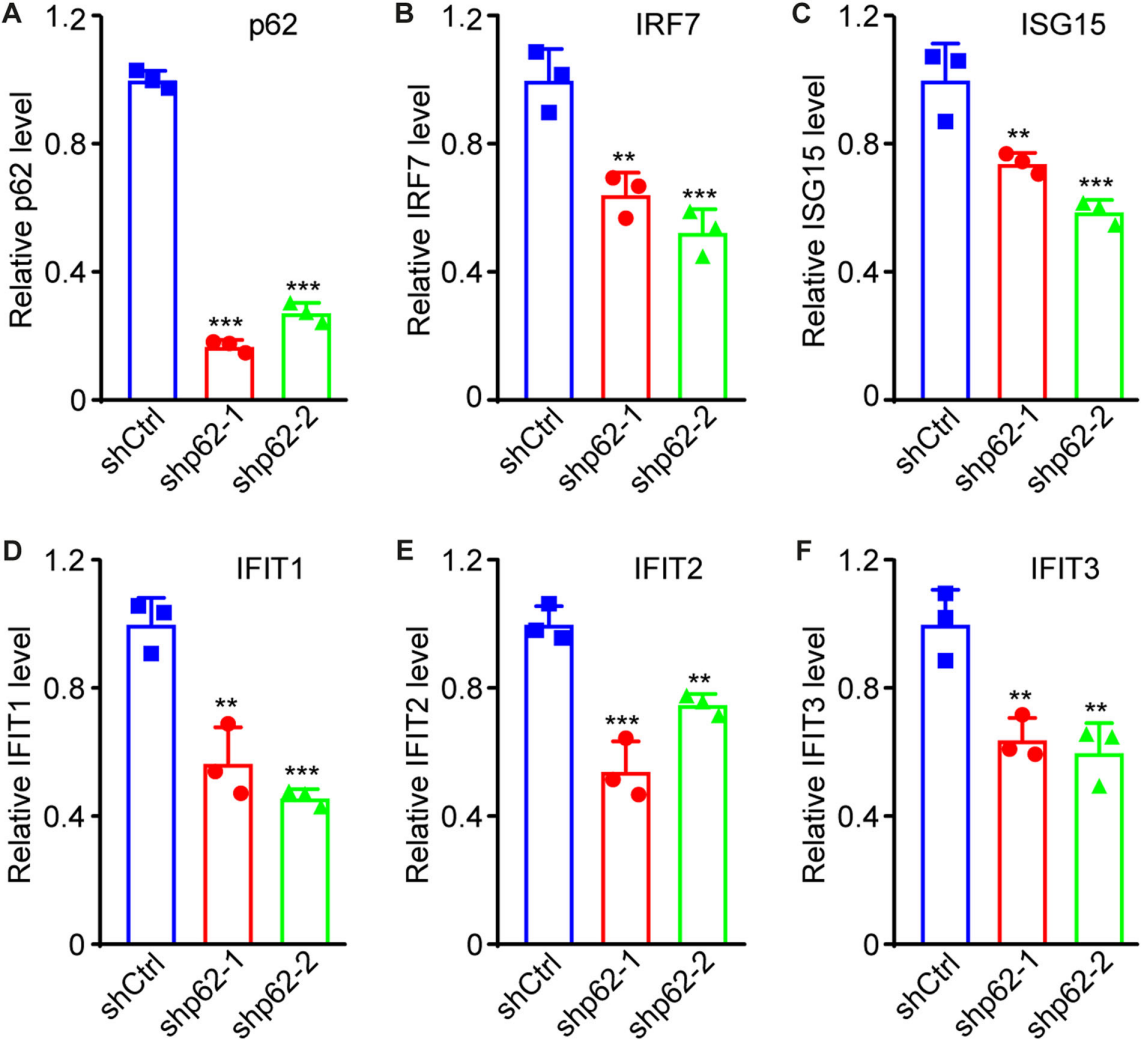
Knockdown of p62 reduces the expression of innate immune-related genes during KSHV primary infection. **A**–**F** HUVEC with p62 knockdown were infected with KSHV for 48 h. Cells were collected for RNA extraction and examined for the expression of p62 (**A**), IRF7 (**B**), ISG15 (**C**), IFIT1 (**D**), IFIT2 (**E**), and IFIT3 (**F**) by RT-qPCR. *, **, and *** indicate *P* values of < 0.05, < 0.01, and < 0.001, respectively; NS not significant.

To investigate p62’s role in innate immune response, we infected HUVEC with KSHV following p62 knockdown. At 48 hpi, p62 knockdown was confirmed (Fig. 7A). p62 knockdown significantly reduced levels of IRF7, ISG15, IFIT1, IFIT2, and IFIT3 genes (Fig. 7B–F), suggesting that p62 was required for maximal activation of the innate immune response during KSHV primary infection.

**Fig. 8:**
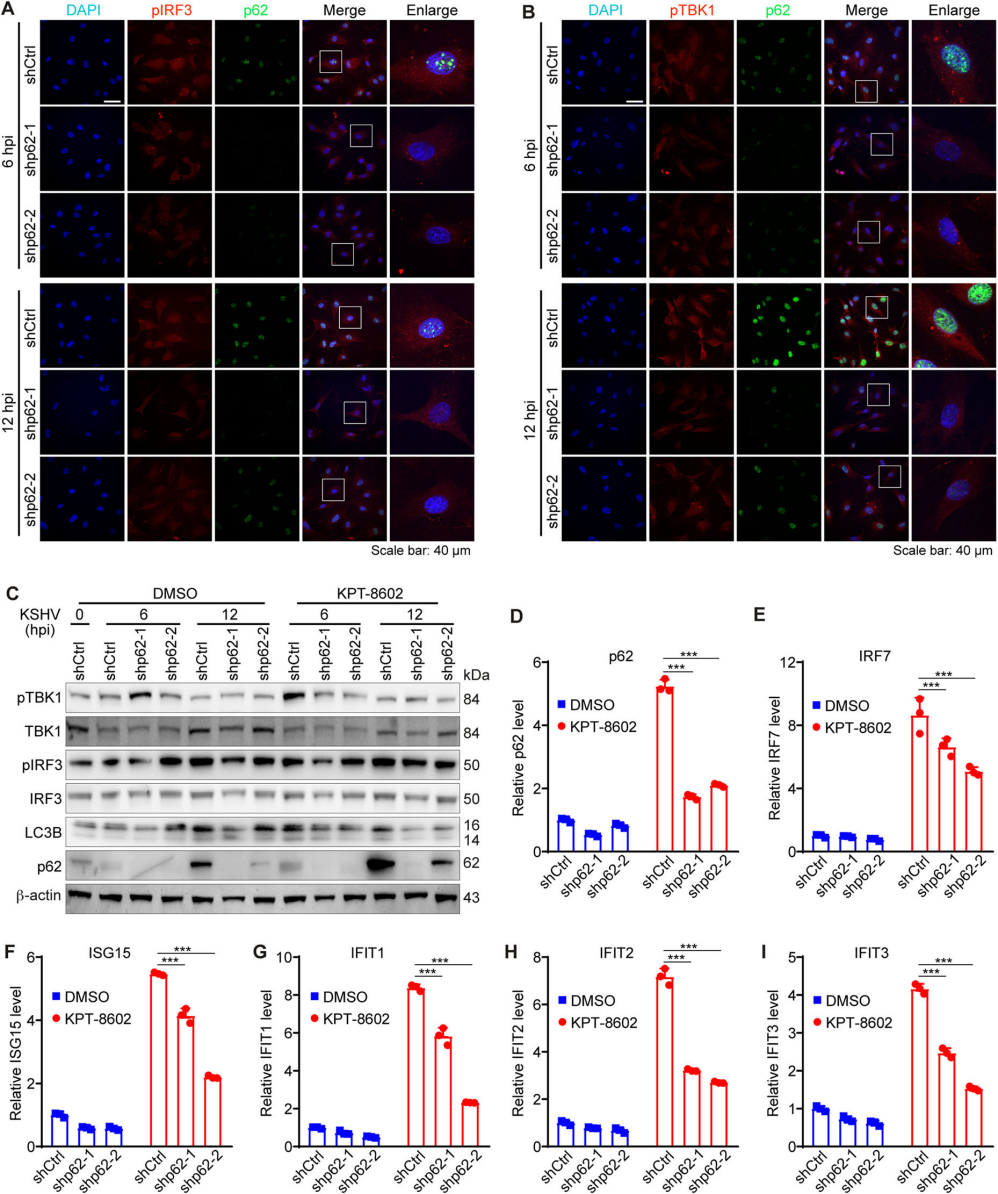
p62 nuclear retention mediates induction of innate immune responses and inhibits KSHV lytic replication during primary infection. **A**, **B** HUVEC with p62 knockdown were pretreated with KPT-8602 at 0.5 µM for 1 h and then infected with KSHV for 6 or 12 h in the presence of KPT-8602. Cells were fixed and co-stained for pIRF3 (**A**) or pTBK1 (**B**) together with p62. Nuclei were stained with DAPI. Images were taken with a confocal microscopy (magnification ×600). Magenta p62 was presented as green pseudocolor for better observation. **C** HUVEC with p62 knockdown were pretreated with KPT-8602 at 0.5 µM or control vehicle DMSO for 1 h, and then infected with KSHV for 6 or 12 h in the presence of KPT-8602 or DMSO. Cells were collected and analyzed for levels of pTBK1, TBK1, pIRF3, IRF3, and LC3B by Western-blotting. β-actin served as the loading control. **D**–**I** HUVEC with p62 knockdown was pretreated with KPT-8602 at 0.5 µM or DMSO for 1 h and infected with KSHV in the presence of KPT-8602 or DMSO. Cells were collected for RNA extraction at 48 hpi and examined for the expression of p62 (**D**), IRF7 (**E**), ISG15 (**F**), IFIT1 (**G**), IFIT2 (**H**), and IFIT3 (**I**) by RT-qPCR. *, **, and *** indicate *P* values of < 0.05, < 0.01, and < 0.001, respectively; NS not significant.

To explore the role of p62 nuclear accumulation in innate immune response upon XPO1 inhibition, HUVEC with p62 knockdown were pretreated with KPT-8602 at 0.5 µM for 1 h and infected with KSHV for 6 or 12 h. Both pIRF3 and pTBK1 levels were reduced in HUVEC with p62 knockdown compared to cells transduced with the scrambled control (Fig. 8A, B). We observed increased levels of pTBK1, pIRF3, and p62 after KPT-8602 treatment in KSHV-infected HUVEC transduced with the control shRNA. However, p62 knockdown reversed levels of pTBK1 and pIRF3 (Fig. 8C). LC3B cleavage was not increased in HUVEC treated with KPT-8602 (Fig. 8C). These results indicated that p62 nuclear accumulation caused by XPO1 inhibition enhanced TBK1 and IRF3 activation. Finally, RNA was extracted and qRT-PCR was performed at 48 hpi to detect p62 and immune-related genes. Treatment of 0.5 µM KPT-8602 increased mRNA levels of p62, IRF7, ISG15, IFIT1, IFIT2, and IFIT3 (Fig. 7D–I). However, p62 knockdown significantly reversed levels of immune-related genes induced by XPO1 inhibition (Fig. 7D–I). Taken together, these results indicated that XPO1 inhibition caused p62 nuclear accumulation, which enhanced type I IFN signaling and blocked KSHV replication.

## Discussion

Viruses often hijack cell machinery to facilitate infection. XPO1 mediates the replication of numerous viruses by regulating the nuclear export of viral and cellular proteins^17,18^. KSHV LANA2 and ORF45 harbor NESs and are regulated by XPO1^42,43^. Nuclear shuttling of KSHV proteins could implicate important functions of importins and exportins in KSHV infection. Nevertheless, XPO1’s role in KSHV primary infection remains unknown.

We show that XPO1 inhibition by KPT-8602 or shRNA-mediated knockdown reduces KSHV replication during primary infection without affecting virus entry and trafficking. Numerous XPO1 inhibitors are available including LMB, ratjadone A, thymoquinone, plumbagin, and piperlongumine^18,44–48^. Recently, a novel class of SINE compounds has been identified^21–23^. Unlike LMB, which permanently binds to XPO1 NES groove, SINE compounds such as KPT-330 (selinexor), KPT-335 (verdinexor), KPT-185, and KPT-350 form a reversible covalent bond with the cysteine 528 residue, and hence are less cytotoxic than LMB^21,22,49^. SINE compounds reduce replication of influenza virus, Venezuelan equine encephalitis virus, and respiratory syncytial virus^22,49,50^. As the second generation of SINE, KPT-8602 manifests improved in vivo efficacy and tolerability in hematologic malignancies compared to KPT-330^24,51,52^. We demonstrate KPT-8602 as a promising therapeutic agent against KSHV replication. Furthermore, our previous study has identified KPT-8602 as an effective agent of KSHV-transformed cells^31^.

Our results show that XPO1 inhibition does not affect KSHV entry, trafficking, infectivity, and expression of latent gene LANA, rather it inhibits the expression of lytic genes. ORF45 is an IE gene-regulating lytic replication^53^. However, the negative effect of XPO1 inhibition on lytic replication is unlikely due to the inhibition of ORF45 nuclear shuttling or ORF45’s direct participation in lytic replication as an expression of RTA, which is required for the expression of other downstream lytic genes including ORF45, precedes that of ORF45^4^. Instead, we observe that XPO1 inhibition leads to p62 nuclear accumulation, and enhanced innate immune response accompanying increased expression of innate immune-related genes including IFIT1, IFIT2, and IFIT3 known to inhibit KSHV lytic replication^41^. Nevertheless, since encapsidated ORF45 protein inhibits innate immune response^38^, it remains possible that XPO1 inhibition might affect encapsidated ORF45 protein function, leading to enhanced innate immune responses and decreased KSHV lytic replication.

The effect of KSHV replication on autophagy remains controversial. RTA overexpression induces autophagy in 293 T cells^54^. While chemical induction of KSHV lytic replication increases autophagy in PEL cells, it is blocked at a late stage of viral replication^55^. Interestingly, KSHV inhibits autophagy by activating mTORC1 during nutrient-deprived conditions, which is important for viral replication^56^. We did not observe any LC3II and p62 changes, indicating that there was no effect on autophagy initiation and flux during the KSHV primary infection of HUVEC. Different cell types and experimental systems likely contribute to these discrepancies.

Autophagy adaptor protein p62 is involved in multiple cellular processes, including autophagy, inflammatory responses, and redox homeostasis^57–61^. p62 dysfunction is associated with Parkinson’s disease, Alzheimer’s disease, and tumorigenesis^62–65^. Interestingly, p62 is categorized as a new group of innate immunity receptors^66^. p62 delivers specific cytosolic components and ubiquitinated targets to autophagic organelles for removal^67,68^. The role of cytosolic p62 has recently been expanded. p62-mediated autophagy selectively degrades RIG-I and STING, attenuating type I IFN signaling^32,33^. p62 harbors active nuclear import and export signals and shuttles continuously between the nucleus and cytoplasm^69^. Nevertheless, p62’s role within the nucleus remains unknown. Our results show that XPO1 inhibition enhances the activation of TBK1 and IRF3 and innate immune responses. While p62 nuclear retention following XPO1 inhibition might lead to reduced cytosolic p62 and enhanced innate immune response^32,33^, there is no change of STING. Hence, the enhanced innate immune responses following XPO1 inhibition are unlikely due to the compromised cytosolic function of p62, rather it might be due to the gain-of-function of nuclear p62, leading to an enhanced innate immune response and inhibition of KSHV replication. Hence, similar to p62, XPO1 appears to function to control activation of the innate immune response in normal conditions. This function is likely important for controlling hyperactivation of the innate immune system in conditions without pathogen invasion. Indeed, genetic mutations of both p62 and XPO1 are associated with various diseases including inflammatory diseases^70–72^. Hence, precaution should be taken when targeting p62 and XPO1 for therapy. It remains unclear how XPO1 inhibition and nuclear p62 enhance innate immune responses. Furthermore, p62 and XPO1 might be directly involved in the infection and replication of other viruses, which might warrant further investigations.

## Supplementary information

Supplemental Materials
