# Supplementary material for: Targeting XPO1 enhances innate immune response and inhibits KSHV lytic replication during primary infection by nuclear stabilization of the p62 autophagy adaptor protein
Source: Cell Death Dis. 2021 Jan 4;12(1):29. doi: 10.1038/s41419-020-03303-1 (PMC7790339; doi:10.1038/s41419-020-03303-1)
Supplement: Supplementary file 1 — Supplemental Materials [file 41419_2020_3303_MOESM1_ESM.pdf]

Supplementary Materials

Supplementary Table S1

Table S1. Primers used in the study

Primer name	Primer sequences (5'->3')
β-actin-F	AGAGCTACGAGCTGCCTGAC
β-actin-R	AGCACTGTGTTGGCGTACAG
LANA-F	TTCCAGTTACCCACCCAAAA
LANA-R	TTGGATCTCGTCTTCCATCC
RTA-F	CACAAAAATGGCGCAAGATGA
RTA-R	TGGTAGAGTTGGGCCTTCAGTT
ORF45-F	GCTTTGCGGCTTAAGTTTGG
ORF45-R	CGCCTCCTCTGGTAGCGA
PAN RNA-F	CGACAAAGTGAGGTGGCATTT
PAN RNA-R	CCGCACACCACTTTAGTCCAA
vIL-6-F	TAAAAAGCTCGCCGATGGCT
vIL-6-R	ACTGATTTTCCAAACTCCGTCG
ORF57-F	AGGTCCCCCTCACCAGTAAA
ORF57-R	GAGGACGTGTGTTTTGACCG
ORF59-F	CGAGTCTTCGCAAAAGGTTC
ORF59-R	AAGGGACCAACTGGTGTGAG
ORF65-F	ATATGTCGCAGGCCGAATAC
ORF65-R	CCACCCATCCTCCTCAGATA
K8.1-F	AAAGCGTCCAGGCCACCACAGA
K8.1-R	GGCAGAAAATGGCACACGGTTAC
vIRF3-F	TGGTCTTCTCCGATGCTTCT
vIRF3-R	TCACCTACACAGTGGGTCATCAC
K12-F	TTCATGTCCCGGATGTGTTA
K12-R	TAATCGCCAACAGACAAACG
vCyclin-F	CATTGCCCGCCTCTATTATCA
vCyclin-R	ATGACGTTGGCAGGAACCA
IRF7-F	TGGTCCTGGTGAAGCTGGAA
IRF7-R	GATGTCGTCATAGAGGCTGTTGG
ISG15-F	ATGGGCTGGGACCTGACG
ISG15-R	GCCAATCTTCTGGGTGATCTG
IFIT1-F	GGAATACACAACCTACTAGCC
IFIT1-R	CCAGGTCACCAGACTCCTCA
IFIT2-F	GGGAAACTATGCCTGGGTC
IFIT2-R	CCTTCGCTCTTTCATTTTGGTTTC
IFIT3-F	TGAGGAAGGGTGGACACAACTGAA
IFIT3-R	AGGAGAATTCTGGGTTGTTGGGCT
p62-F	ATCAGCTTCTGGTCCATCGG
p62-R	ATCGATATCAACTTCAATGCCCAG

Supplementary Figure legends

Fig. S1 Cytotoxicity of KPT-8602 to HUVEC. (**A-D**) HUVEC were treated with the indicated doses of KPT-8602 for 24, 48, 72 and 96 h. The cell viability was analyzed by MTT assay. *, **, and *** indicate P values of <0.05, <0.01, and <0.001, respectively; NS, not significant.

Fig. S2 XPO1 inhibitor KPT-8602 treatment induces p62 nuclear retention. HUVEC were pre-treated with 0.5 µM KPT-8602 or vehicle control DMSO for 1 h and then infected with KSHV for 12 h in the presence of KPT-8602 or DMSO. Cells were fixed and stained for p62. Nuclei were stained with DAPI. Images were taken with a confocal microscopy (magnification ×600).

Fig. S3 XPO1 knockdown induces p62 nuclear retention. HUVEC with XPO1 knockdown were infected with KSHV for 12 h. Cells were fixed and stained for p62. Nuclei were stained with DAPI. Images were taken with a confocal microscopy (magnification ×600).

Figure S1

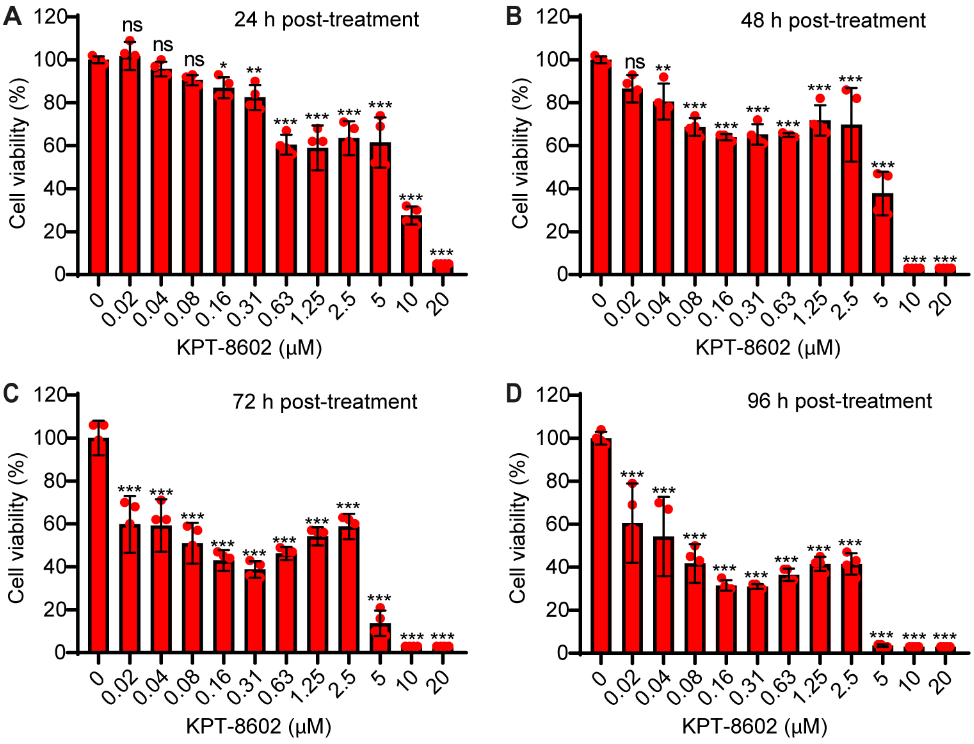

A
24 h post-treatment
B
48 h post-treatment
C
72 h post-treatment
D
96 h post-treatment
Cell viability (%)
KPT-8602 (μM)
0
0.02
0.04
0.08
0.16
0.31
0.63
1.25
2.5
5
10
20
ns
*
**
***

Figure S2

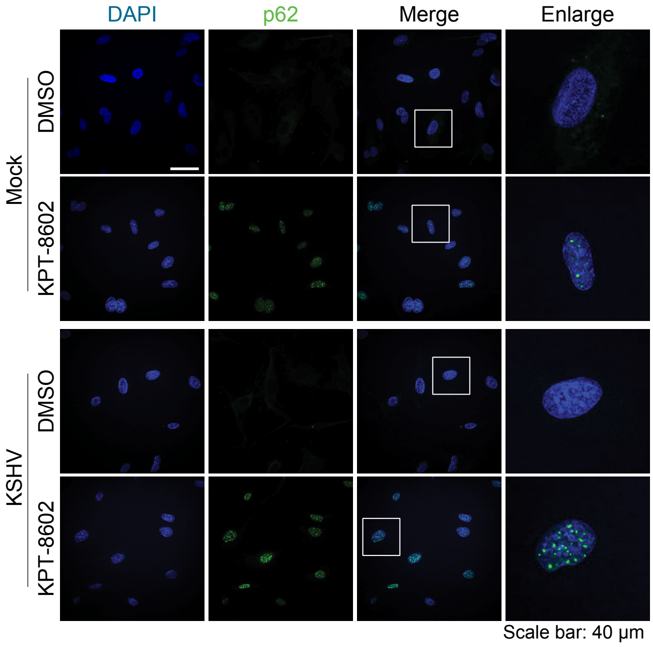

DAPI
p62
Merge
Enlarge
Mock
DMSO
KPT-8602
KSHV
DMSO
KPT-8602
Scale bar: 40 μm

Figure S3

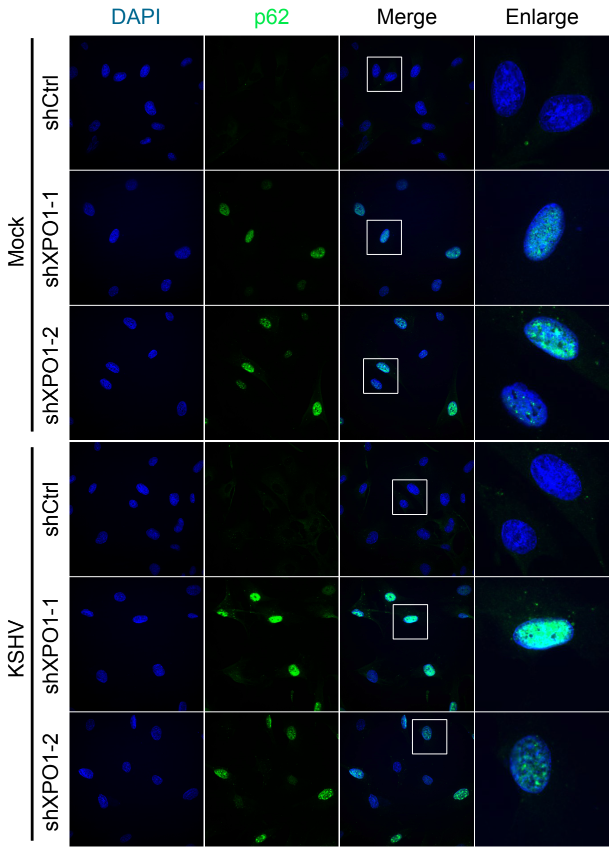

DAPI
p62
Merge
Enlarge
Mock
shCtrl
shXPO1-1
shXPO1-2
KSHV
shCtrl
shXPO1-1
shXPO1-2
